# Examination of social worlds of risky drinking. Insights from Twitter data analysis

**DOI:** 10.1371/journal.pone.0289237

**Published:** 2024-02-23

**Authors:** Ashir Ahmed, Jenny Martin, David Towl, Zac Haussegger, Muhammad Babar

**Affiliations:** 1 College of Computer and information Systems, Prince Sultan University, Riyadh, Saudi Arabia; 2 Department of Media and Communication, Swinburne University of Technology, Melbourne, Victoria, Australia; 3 Department of Social Sciences, Federation University, Victoria, Australia; 4 Access Heath and Community Health, Hawthorn, Victoria, Australia; La Trobe University - Melbourne Campus: La Trobe University, AUSTRALIA

## Abstract

Rich nature of social media data offers a great opportunity to examine social worlds of its users. Further to wide range of topics being discussed on social media, alcohol-related content is prevalent on social media and studies have found an association between this content and increased consumption of alcohol, cravings for alcohol and addiction. This study analyses social media data to examine social worlds of risky drinking in Victoria, Australia. This study conducted a scoping literature review and two online surveys, one with the general community and the other with health professionals, to determine key words to search for on social media sites. These keywords were used in a social media analytics tool called Talkwalker to generate quantitative and qualitative data on the social media users and their conversations. NVIVO was used for developing categories and themes in a sample of 172 posts. A total of 1,021 results were obtained from Twitter. The main demographic group found to be involved in conversations about drinking alcohol on Twitter was young fathers aged 25–34 years. The culture of alcohol consumption in Victoria for Twitter users is reflective of Australia’s drinking culture within which risky drinking, and in particular binge drinking, is normalised.

## Introduction

Drinking alcohol is undoubtedly a part of Australian culture with alcohol freely available and consumed at most social events. People may drink alcohol for the physical effects of enhanced pleasure or reduced anxiety as well as for a sense of belonging and identity [1, 2]. Drinking alcohol is considered the norm with risky drinking and alcohol-related harms more likely to be associated with others who may be considered in need of professional help. Social tolerance of intoxication can lead to excuses and acceptance of undesirable behaviour and associated alcohol-related harms [1]. This is culturally determined according to group expectations of alcohol consumption and views on intoxication [2]. In recent years binge drinking has become part of youth culture with regular heavy drinking the norm. In Victoria (Australia), the location of this study, individual and peer group cultural change interventions are targeted at 16-29-year-olds who are considered to be at highest risk of alcohol misuse including binge drinking [3].

### Risky drinking

Alcohol misuse is risky drinking that has not yet reached the point of dependence but is causing difficulties in a person’s life and/or those associated with them [4]. Misuse generally occurs when drinking is used as a coping mechanism. A person misusing alcohol may feel a need to drink for relaxation or to improve mood and may hide, or lie about, drinking habits. Binge drinking is when intoxication levels are reached in a couple of hours often by drinking shots and playing drinking games [5]. Risks associated with binge drinking are violence, reckless behaviour, unprotected sex, driving over the legal limit, loss of valuables, memory loss, reputational and relationship damage, and alcohol poisoning [6].

This study was conducted during the COVID-19 pandemic that saw a state of emergency declared in Victoria and stringent lockdown restrictions. The federal government introduced social distancing measures that prevented people from consuming alcohol at licensed premises. Australian states and territories, including Victoria, responded by allowing authorised licensed premises to retail alcohol for home delivery and take-away. This facilitation of access to alcohol was in the context of families at home together in lockdown with increased psychological and financial pressure due to the pandemic [6]. This action was contrary to the advice of the World Health Organisation advising governments to limit alcohol consumption during lockdown due to increased vulnerability and risk of health and mental health issues, violence and risky behaviours [7].

### Social media

Social media is saturated with content related to alcohol that is influencing drinking habits and cultures [8]. This content, for the most part appears harmless, yet research studies suggest otherwise, as there is a relationship between the posting of alcohol-related content and increases in alcohol cravings, consumption and dependence [9, 10]. In 2020, just under three quarters (71%) of the Australian population (25,499,884) used social media with 85% of these users 13 years and older and most using mobile devices. During November 2020, the first month of the study period, the social media sites with most active users by Australians were Facebook (16,500,000), YouTube (16,000,000), Instagram (9,000,00), LinkedIn (6,500,000), Snapchat (6,400,000), Twitter (5,800,000), WeChat (2,900,000), TikTok (1,100,000) and Pinterest (290,000) [11]. Most Australian social media users are aged 18–34 years (76.5%). This is followed by 35–44 year-olds (12.5%), 13–17 year-olds (5.5%), 45–54 year-olds (4.3%) and 55–64 year-olds (1.2%) [12]. The study findings overwhelmingly pertain to Twitter users. Twitter use in Australia reflects global trends of users who are mostly male, well-educated with an above-average income and urban dwelling. Australian Twitter users are aged between 14–24 years (38%), 25–34 years (34%), 35–49 years (29%) and 50 years and over (19%) (Roy Morgan, 2016, p.2).

## Research design

The aim of this exploratory study is to find out who engages in conversations about alcohol use on social media and the main topics and themes of these conversations. We were particularly interested in risky drinking conversations. This study aims to address the following research question:

What social media conversations do people between the age of 16-40-year-olds engage in about drinking alcohol?

In Victoria (Australia), the location of this study, people between the age of 16- and 29-year-olds are at highest risk of alcohol misuse including binge drinking.

### Data collection

Data collection was conducted using Talkwalker, a social media analytics platform leveraging artificial intelligence to monitor and analyse real-time online conversations across diverse social networks, news websites, blogs, and forums in numerous languages. In the context of this study, Talkwalker’s role involved utilizing the search terms provided by our surveys, in conjunction with insights gleaned from a literature review conducted by our research team, to pinpoint the social contexts where users employed these terms during their online interactions. Moreover, Talkwalker not only scrutinized qualitative social media content, encompassing posts, tweets, and comments, but also explored various demographic facets of users engaged in online communication.

Talkwalker’s monitoring capabilities facilitated searches across publicly accessible data on various social media platforms. It retrieved items matching specific search criteria, encompassing search terms, preferred timeframes, geographical regions, languages, media formats, and even device usage. Alongside each data point, Talkwalker furnished additional information, including user demographics, post sentiments, and thematic context. It’s essential to emphasize that the data collection process by Talkwalker adhered to the privacy policies of the respective social media platforms from which the data originated. Talkwalker demonstrated proficiency in analysing multiple parameters associated with a user’s profile when identifying keywords within their posts. These parameters encompassed details such as location, age, occupation, gender, and interests, which could either be explicitly available on a user’s public profile or inferred from their ’bio’ or the pages they followed.

These demographic dimensions yielded insights into the gender, age, social and familial status, interests, and geographic locations of individuals constituting the social realms related to risky drinking culture. Information regarding location, age, occupation, gender, and interests was either explicitly found on users’ public profiles or inferred from their ’bio’ or the pages they followed. Geographic location for data collection was specified as Victoria, Australia. Our study received approval from the Research Ethics Committee at Swinburne University of Technology (Australia) under Review Reference: 20202944–4889. All human participants provided written informed consent before participating in the study.

It’s pivotal to acknowledge that when analysing Talkwalker’s global data summaries, sample sizes for each parameter may exhibit variations. This divergence arises because some users’ profiles may offer information about gender but not occupation, while others may include occupation data but lack gender information. It’s crucial to note that our study operated within the functional constraints provided by Talkwalker.

### Data analysis

Data analysis was conducted in two stages. In stage 1, the initial search terms derived from the scoping review and surveys were entered as a pilot search on Talkwalker returning 4,714 results. Terms that did not refer to drinking alcohol were identified in a random sample of 200 results. Two authors independently and then jointly reviewed these terms. Following this data cleaning, the final search terms used the Boolean operator AND NOT to limit the collection of irrelevant data resulting in a final search yielding 1,021 results.

In stage 2, an additional random sample of 200 results was taken from the revised dataset to reassess the results for their relationship to drinking and to identify main themes related to drinking alcohol. Of the 200 results, 172 were found to have search terms used within a drinking context. This was deemed an appropriate end point of the topic query, as greater than 80 per cent of results were now relating to drinking alcohol. These 172 posts were then analysed and coded using Braun and Clarke’s [13] thematic analysis methodology. In terms of the decisions that needed to be made regarding the type of analysis performed, an inductive, rather than theoretical, reading of the data was preferred, to identify new themes emerging from the results. Additionally, in terms of depth of analysis, the data was read on the semantic level and then interpreted by the research team [13]. The handling of these 172 posts was done using the qualitative data analysis software NViVO, which stored the codes that emerged from the data. Analysis was performed on these results on three different occasions. First, the data was read through and familiarised by one author. Next, the results were coded as to the themes that were within each post. Lastly, the process was repeated to apply learnings from the first round of coding onto all the results. Saturation was reached with no further themes arising in the data. A second author also coded the data into themes with consensus reached between both authors.

## Results

### Talkwalker demographic data

**Fig 1:**
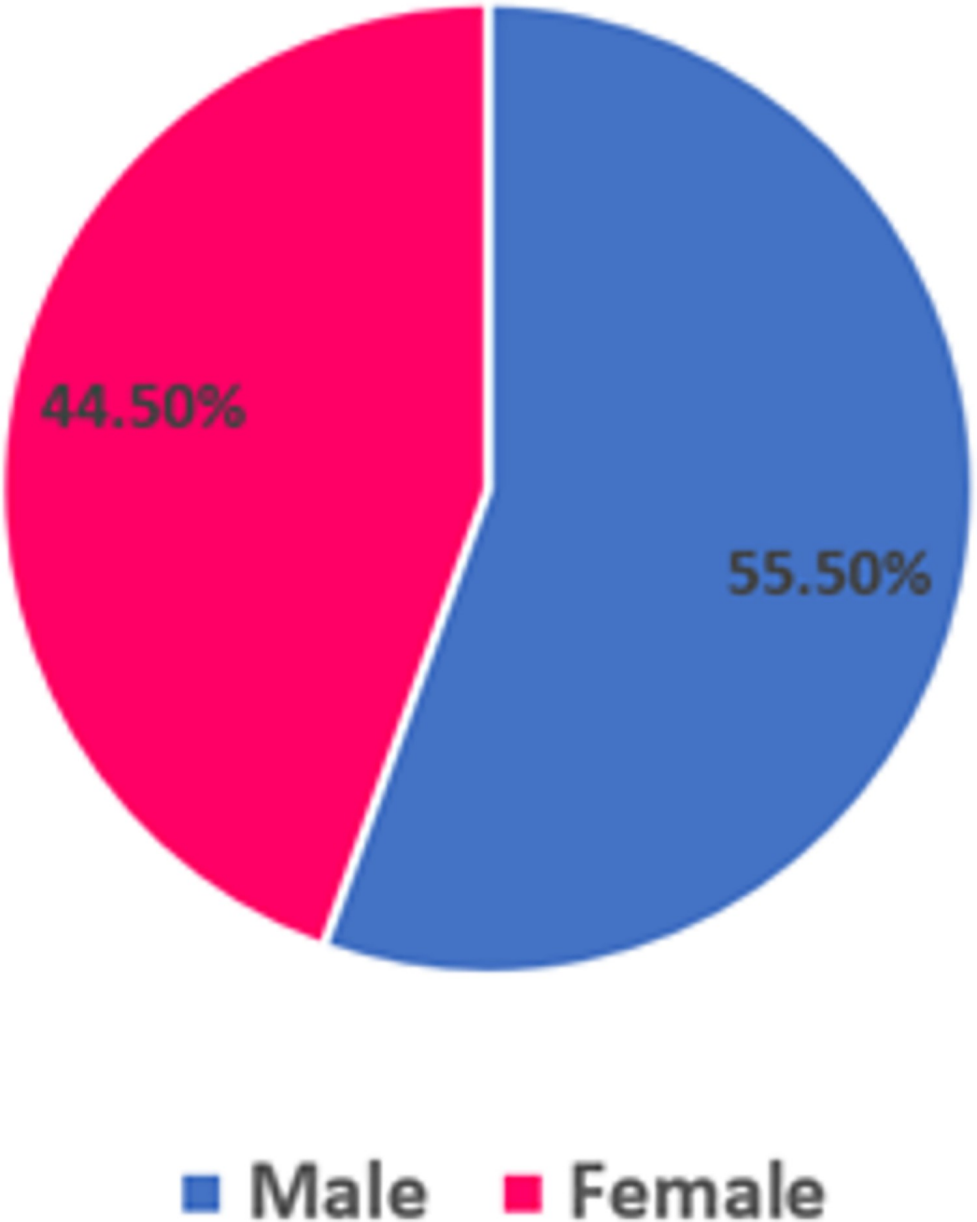
Data on gender.

**Fig 2:**
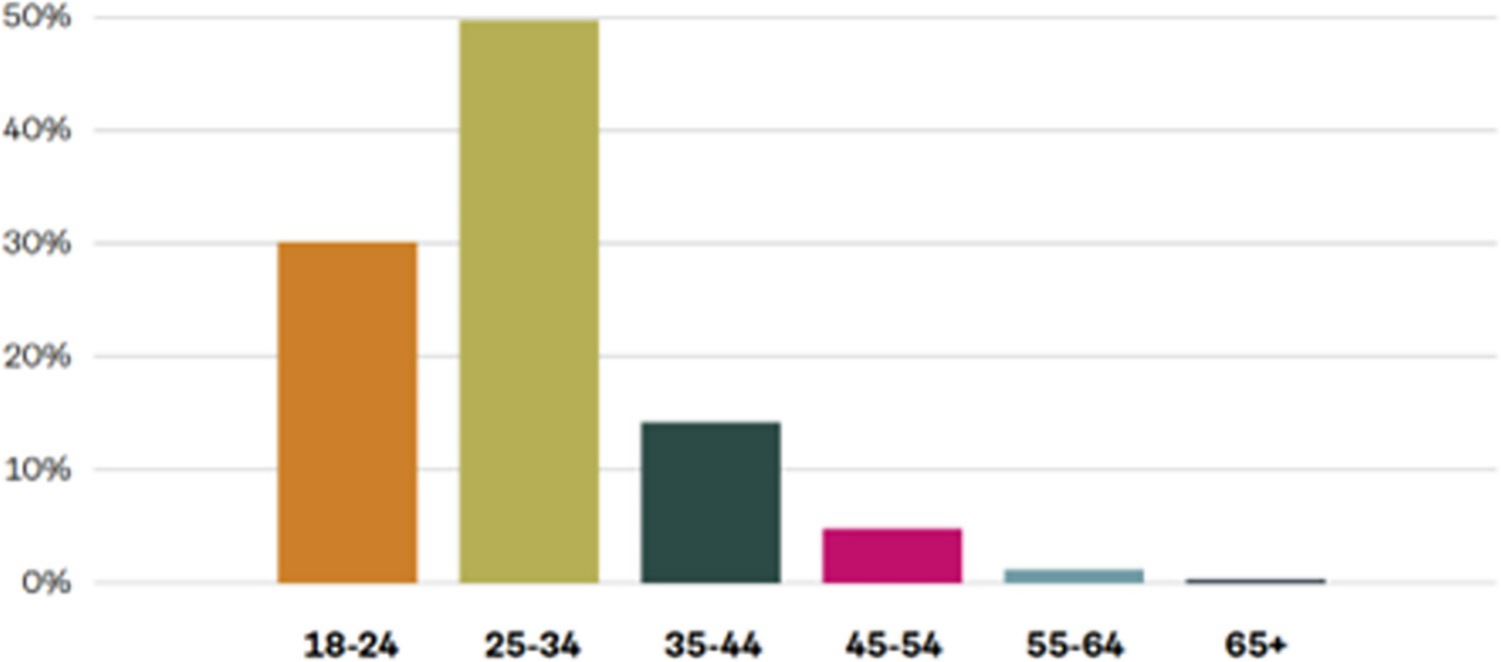
Data on age groups.

The majority of the 1,021 results retrieved by Talkwalker were made by males (55.5%) who were mostly aged 25–34 years (49.7%) followed by 18–24 years (30.1%), 35–44 years (14.2%), 45–54 years (4.8%) and 65 years and over (1.2%). Gender data was collected on 86.97% (n = 888) of Talkwalker results with age data collected on 56.61% (n = 578) results as shown in Figs 1 and 2.

**Fig 3:**
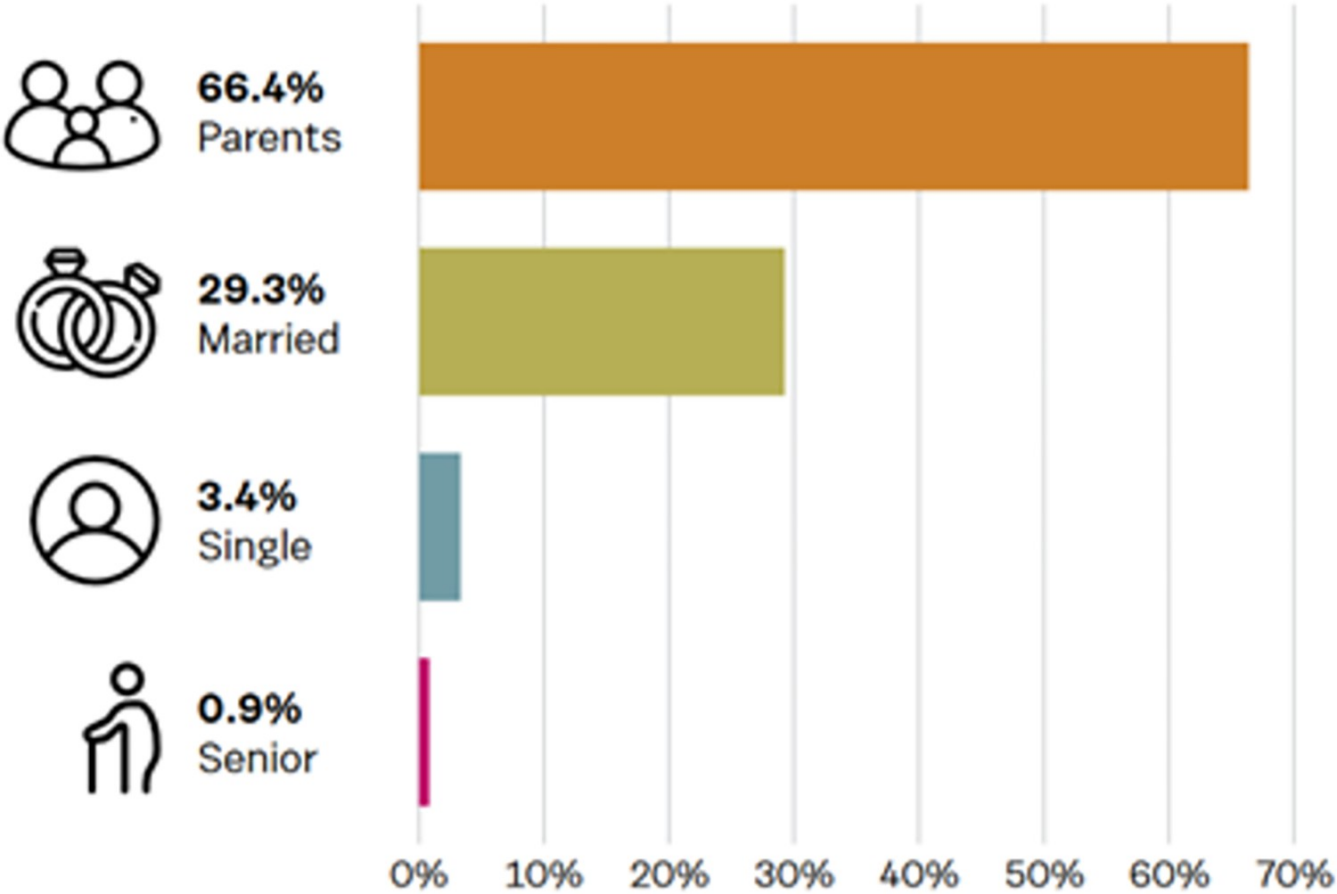
Data on family status.

**Fig 4:**
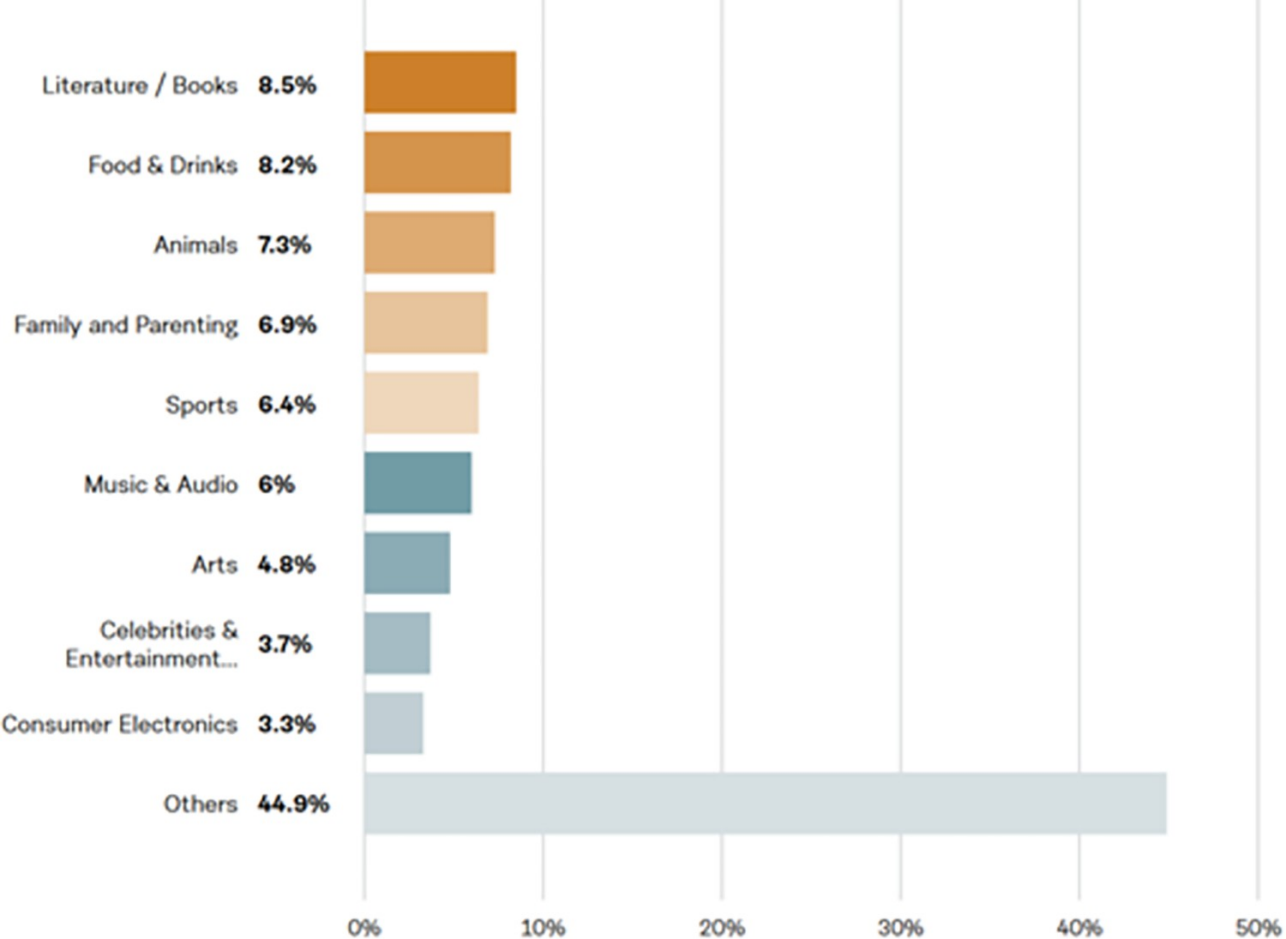
Data on profession.

Of the 11.36% (n = 116) results for family status, ‘*parents’* (66.4%) were the highest followed by *‘married’* (29.3%), *‘single’* (3.4%) and *‘senior’* (0.90%). These results are confounded by the potential of overlapping categories such as a *‘married person’* who is a ‘*parent’* and a *‘senior’*. This low-level response rate is not representative of the entire dataset. The main occupation identified by Talkwalker from 39.7% (n = 405) of results was *‘author/writer’* (18.3%) followed by *‘photographer’* (6.6%), *‘artist’* (5.4%), *‘musician’* (5.4%), *‘journalist’* (4.7%), *‘entrepreneur’* (4.2%), *‘teacher’* (3.5%) and *‘scientist’* (3.2%) as shown in Figs 3 and 4.

### Main themes

The main themes identified in 172 tweets from the final dataset were: ‘advertising’, ‘binge drinking’, ‘observations of intoxicated people’, ‘COVID-19 lockdown’, ‘celebrations and entertainment’.

### Advertising

The conversation among the selected group and people and relevant retailers was reported in this section. Forty-five tweets were ***advertisements*** / ***promotion of events or alcohol products*** / ***drinks by organisations***, ***alcohol retailers***, ***travel and tourism***, ***social groups***, ***food and drink venues*** and ***individuals***. Some tweets from advertisers mimicked tweets from individuals, public figures, and organisations. Frequently mentioned terms were: *‘happy hour’*, *‘wine time’*, *‘beer time’*, *‘Twitter drinks’* and *‘virtual wine tasting’*. Tweets were made by breweries, wineries and restaurants (10), alcohol retailers (7), celebrities, influencers and educators (5), an alcoholics anonymous regional and rural branch (4), individuals (4) unidentified sources (6), tourism operators (5), a coffee drinking club (1), a women printers’ organisation (1), a corporate association (1) and a comic readers club (1).

Tweets recommended drinking as a scheduled or regular activity: *“It’s #notfriday but who cares 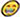 #wineandcheese #afterdinnerdrinks #cheers”* (T185); *“Standard Time to get heavily inebriated very soon 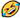
” (T154); “It’s always wine o’clock”* (T188). It is likely that the use of happy hour in advertisements for an Alcoholics Anonymous event is a play on words. A holiday park business tweeted: “Camping happy hour done right. 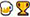
” (T145). Tweets commented on venues: *“Yes*, *mistakes were made*, *but it also offered $6 pints in happy hour and that kind of value needs to be acknowledged”* (T186). Faster and better service was wanted during happy hour. Some tweets included ingredients for making drinks: *“Next time you think you’re too shitfaced to make #cocktails, try XXXX Watermelon Daiquiri. You’re welcome. 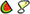
”* (T9).

A retailer tweet appeared to be a personal communication: *“When acts of kindness turn into Gin! 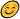 thanks”* (T164). A tweet from a business for a drinking game appeared to be from an individual: *“I just earned the ‘Happy Hour Hound’ badge”* (T85). A tweet from a personal account mimicked a public relations account from a government health and human services department: *“Hair of the dog time? 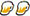 Keep this party rolling! 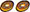
”* (T33).

### Binge drinking

Thirty-seven tweets suggested active ***binge drinking*** or ***wanting to drink***. Drinking was to get tipsy (7) or legless/drunk/shitfaced (8), and for mental wellbeing/to ease pain (4). Other tweets mentioned individual responsibility for actions (7), effects of alcohol (4), lost days due to drinking (2), daytime drinking (2) meeting people (2), and drink driving (1).

A tweet mentioned drinking on an empty stomach: *“Am I nearly tipsy off 2 cruisers and half a bottle of wine on an empty stomach*? *Maybe”* (T22). A tweet suggested using other drugs as well: *“Anyone wanna come over tonight*, *get tipsy*, maybe h*gh, *put on make-up and chill out to 80’s pop classic*?*”* (T11). Drinking with the intention of getting drunk included: *“It’s a dangerous day to be a beer*, *I just wanna get pissed”* (T133). A writer worked while intoxicated: *“My writing process is generally getting a bit inebriated*, *staying up way too late*, *and just blurting onto a page*. *Then editing in the light of a sober day*. *It does not always result in gold”* (T170). Mental health and well-being tweets were: *“Since I’m wfh like forever now, it’s ok to sometimes start happy hour at 3:30-ish…isn’t it? Just for mental health purposes. I mean, look at the world ffs—some days you just need it! 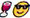
”* (T25) and *“just get tipsy enough to either dull the pain or enhance euphoria”* (T188).

Tweets on responsibility for own actions when intoxicated were: *“I accept this responsibility with a heavy and tipsy heart”* (T49) and *“Summoning this day to come faster. 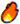 I’m a bit tipsy so don’t take me seriously ☺☺”* (T29). A tweet referred to media responsibility: *“That is how our whole media cycle operates*. *Clear out trash on Friday arvo*, *majority get pissed every weekend and forget*, *then the Australian sets a fresh agenda on Monday*. *Rinse & repeat”* (T44). A tweet suggested craving for alcohol: *“very unfair that i am currently "very pregnant" and "not allowed" to get shitfaced”* (T65).

Tweets mentioned effects of drinking and social aspects: *“I did mine with a bunch of other people*, *and we were all super hungover because we got pissed the night before*. *That’s the way it’s supposed to be done*, *right*?*”* (T4) and lost days from intoxication: *“Just had hair of the dog and it has ruined me*. *Days are gone”* (T197). Daytime drinking tweets included: *“It’s still daylight and I’m already tipsy*. *I blame daylight savings*!*”* (T138) and *“Beer garden straight after brunch*. *Someone’s tipsy at 4pm*” (T96). One tweet glorified drinking*: “Can we all take a minute to remember all the people who were not allowed to get shitfaced before breakfast and perform the walk of shames with shoes in hand today We appreciate your sacrifice 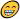
”* (T142).

### Observations of intoxicated people

Thirty tweets commented on observations of ***violence and bullying*** (10), ***reputation*** (3), ***racial stereotypes*** (2), ***mental wellbeing*** (2), ***disinhibited speech*** (5), ***responsibility of others*** (3), ***drink driving*** (2), **curiosity** (2) and ***an intoxicated animal*** (1).

Tweets related to violence against older people and women while intoxicated: *“This is about privileged men in male dominated schools*, *units & workplaces including politics*, *business*, *law*, *military*, *police etc being sexually aggressive*, *inebriated*, *dishonest to partner*, *pretending to be on board about #VAW—So familiar everybloodywhere*!!*”* (T105). The use of intoxication as an excuse for violent actions was questioned: *“XXXX said he was "just really intoxicated" and "wasn’t really thinking"*. Imagine what this monster could do to a woman anytime in the future when he is thinking” (T61). A tweet suggested possible bullying: “*His mates should get him really pissed one night and shave it all off for a laugh 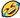
”* (T200).

Some tweets questioned the association between intoxication and violence: *“Its not being drunk that is the issue*, *its being a drunk and an idiot*. *Plenty of happy*, *fun intoxicated people out there that don’t harm others*, *get violent etc*. *punish the person*, *not the blood alcohol level*. *Pretty sure police would be happy to not babysit drunk ppl*.*”* (T115). Tweets questioned the evidence on the harms of intoxication: *“Usually when we’re tipsy we just collapse in a heap*. *Sober’s where we do the MOST damage surprisingly hun*…*”* (T99). Tweets commented disapprovingly of intoxicated women’s reputations: *“I called her an alcoholic last night*, *I think she got pissed and ruined her reputation”* (T183). There were not any similar tweets related to men’s reputations.

Tweets were made on responsibility for criminal acts: *“Does this mean for example that if someone is intoxicated on the street*, *then beats or robs someone (happens) or causes property damage (happens)*, *they can simply claim that they were having a health issue*? *Bigger crimes are stopped early by stopping small incidents”* (T125). Tweets expressed concern for mental wellbeing: “She’s not mentally okay, she shouldn’t be live streaming while intoxicated” (T113) and *“I wonder what XXXX mental health was like when he punched a bar manager for refusing to serve drinks to his pissed mates at the pub one night”* (T144).

A tweet commented on a video of a squirrel drunk on fermented pears that went viral: *“Cannot stop laughing at this. #SQUIRREL 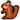
”* (T137). Tweets on drink driving were: *“My cousin was arrested driving at night on the wrong side of the road with no headlights or seatbelt*, *with a BAC of 0*.*15*. *I can’t even imagine how shitfaced you’d be at 0*.*2”* (T199) and *“He dances like a guy trying to drive his car home intoxicated*…*”* (T111).

### COVID lockdown

Twenty-eight tweets referred specifically to drinking during lockdown including ***virtual drinks***, ***Happy Hour***, ***Twitter drinks*, *Zoom drinks***, ***isolation drinks***, ***lockdown and virtual wine tasting*** (16), ***COVID-19 restrictions*** (5), ***intoxicated crowd behaviour*** (2), ***coming out of COVID lockdown*** (2), ***managing in lockdown*** (2) and ***online shopping while intoxicated*** (1) Virtual drinks tweets included: *“Thank you mama! For real, bring on Twitter drinks 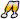 we really deserve it the most, carrying during a pandemic! I’ll be tipsy off the smell 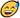
” (T69); “Happy hour Melbourne lockdown drinks time! 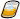 Have a great week-end everyone!”* (T131) and *“Zoom work happy hour yay*!!*”* (T152). A tweet called for responsible crowd behaviour during lockdown: *“Third wave imminent based on the behaviour of intoxicated masses on Swan Street. PUT
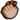
YOUR
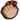
MASK
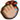
ON”* (T24). A further tweet commented: *“Sure; can’t imagine a group of inebriated people breaking into song and dance at a pub- or when leaving*…*”* (T107). Tweets referred to COVID lockdown restrictions: *“As much as I think sports events are stupid in a pandemic*, *it’s beyond moronic to write articles like this*, *arguing for business over health*. *Thousands of teens getting intoxicated in clubs is not the same as an outdoor sports event”* (T153). Tweets commented on consistency in lockdown restrictions: *“You can’t walk your dog 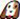 or yourself 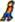 to exercise your legs, but you can go to the bottle shop to get legless 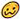
”* (T114). A coming out of lockdown tweet was: *“Looking forward to getting back out amongst it. And when I say it I mean you lovely people. Cheers Big Ears let’s have some Beers! 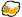
”* (T129). A tweet thanked the Victorian state premier: *“Thanks for your tireless effort and standing strong in the face of fierce adversity for Victorians*, *and indeed*, *all Australians*. *#Cheers #getonthebeers”* (T34).

### Celebrations and events

Twenty-four tweets were for ***celebrations and events*** including ***sport*** (12), ***birthdays*** (5), ***studies*** (2), ***Christmas***, (1) a ***professional World Day*** (1), *end of the working week* (2) and ***reason not specified*** (1). Sports tweets were for two football codes: *“Is it a crime to be too tipsy before the first bounce of an AFL Grand Final*? *Asking for a friend*. *In case XXXX is reading*, *that friend isn’t at mine”* (T194). Racing tweets were: *“Great finish to the day”* showing a photo of a bottle of ale with a racing logo (T52); *“And silly drunk girls who are pissed by Race 4”* (T127) and *“Since 2013, seven horses have died during or after the Melbourne Cup. Hope you enjoyed getting shitfaced and losing your money though 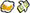
”* (T117).

A birthday tweet was: *“Goin the whole hog 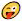 #happybirthday”* (T57). A Christmas tweet recommended an alcoholic drink for the occasion. Tweets on studies suggested alcohol for comfort when preparing for exams and being intoxicated while studying: *“Our swotvac starts tomorrow*. *I have ordered in extra bath bombs and chocolate and wine”* (T12) and *“Fk it I’m looking up cat and mink biology. Im tipsy, but imma do it 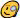
”* (T50). The World Teachers’ Day tweet was: *“End of a big week in #Melbourne town- finally reaching for the top shelf and Happy World Teachers Day- you have been stellar 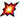
over this long lockdown2 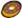
”* (T106) with a photo of near empty bottles of alcohol. An end of the working week tweet was: *“Friday is almost over here & I’m a little tipsy*!*”* (T30). An unspecified celebration tweet mentioned a celebrity: *“Yes*!!! *Happy with you*!!: *XXXX wine time and cake*?*”* (T6).

**Entertainment: Movies, gaming and television.** Eight tweets related to ***movies*** (4), ***gaming*** (3) and ***television*** (1). Tweets mentioned being tipsy while watching a show suggestive that this is a desired state for viewing: *“I will be tipsy*, *I will have two popcorns to myself*, *and I will cackle loudly”* (T159). Movie tweets refer to horror films and binge-drinking: *“Streaming some Blair Witch while kinda tipsy”* (T175) and *“Shot 6 since movie creeper tipsy”* (T160). A television tweet referred to reality dating: *“Is XXXX absolutely shitfaced because there is zero future with the bloke*!*”* (T22). A gaming tweet mentioned effects of alcohol: *“tipsy and playing league of legends”* (T93). A tweet mentioned a video game and film composer: *“I love this tradition of XXXX getting tipsy on nog and engaging with his fans*. *<3”* (T195).

## Discussion

This study collected data from people between 16- and 40-year-olds. However, the study found that the main age group for risky drinking, and in particular binge drinking, is 25 to 34 years and not 16 to 29 years as reported in previous studies [14–16]. Of these risky binge drinkers, most were fathers of dependent children and in relationships. This is significant due to the association between harassment, violence and abuse, and child neglect and intoxication [17–19]. There was clear overlap between themes with advertising and events promotion encouraging binge drinking, drinking games and comfort drinking and suggesting that Victorians deserved to be intoxicated during COVID-19 lockdown [20]. This activity is of particular concern due to the known social harms associated with binge drinking and comfort drinking [1, 21]; The tweets in the advertising, binge-drinking and entertainment themes portrayed getting drunk as an intended state for socialisation, fun, relaxation, creativity and for mental wellbeing. Tweets on sporting events were suggestive of a culture of drinking which is indicative of the need for government intervention to promote responsible social drinking. The association of drinking with sport is likely to be reflective of targeted marketing of alcohol as the participants were not engaged in active or sporting occupations or interests.

Risky behaviours while intoxicated such as seeking random company, driving and preparation for exams were normalised alongside negative impacts of intoxication such as not remembering things, being hung over and lost days. This finding supports the need for the identification of online risky drinking social worlds for personal alcohol health education that is suited to discussion of the risks associated with alcohol misuse, what responsible drinking entails and challenges alcohol-related harmful behaviours [1]. This conversation will be guided by the preferred language and communication style of the social media site.

Social harms or unacceptable behaviours mentioned in the tweets were generally related to observations of others or as an excuse for the person’s own actions consistent with the view that drinking is normalised within the community and within peer groups or social worlds [1]. Tweets on mental health concerns were for both men and women with drinking suggested as a way of coping with COVID-19 restrictions [22]. Oppositional views were expressed on intoxication and crowd behaviour during the COVID-19 pandemic and sporting events and the association between intoxication and violence against women including sexual violence. Interestingly, it was women’s reputations that were presented as of most concern and not men’s, suggestive of an acceptable culture of male drinking. Easy access to alcohol during the pandemic was questioned in light of other restrictions questioning both cultural and commercial motivations by government [23]. Accordingly, further government action is required in accordance with the World Health recommendations to limit rather than increase access to alcohol during a pandemic due to increased health and wellbeing risks and associated harms to self and others [24]).

## Future research and policy considerations

An important consideration when conducting social media research is what data is likely to be accessible publicly on the targeted study population. What was intended as a broad social media analysis of alcohol consumption ultimately ended up being an analysis of activity on one social media platform only. Marketing social media search engines, such as Talkwalker are a potential source of valuable data for social research. However, adjustments are required to the platforms search algorithms for social research purposes. Adjustments of parameters such as geographic settings, selection of search terms, language, and utilization of various social media platforms could be implemented to achieve a more extensive and diverse representation of the population. These adjustments will contribute to the generalizability of research findings. Moreover, future research would benefit from the adjustment of Talkwalker’s algorithm to view risky drinking as negative and responsible drinking as positive. This would enable the research team to then target the risky drinking posts generated by Talkwalker in a more informed way. It is important to be mindful of most social media analysis being conducted primarily for marketing purposes and not for social research. Adjustments to social media analysis search engines need to be made to include gender diversity, LGBTQI+ and same sex attraction. Further research requires careful consideration of the inclusion of search terms with varied meanings that can cloud the data and breach available data limits. Finally, it would be valuable to consider the research findings to policy making regarding the access and consumption of alcohol during pandemic and similar situations.

## Supporting information

S1 Data (XLSX)
